# Identification and validation of heterotypic cell-in-cell structure as an adverse prognostic predictor for young patients of resectable pancreatic ductal adenocarcinoma

**DOI:** 10.1038/s41392-020-00346-w

**Published:** 2020-10-20

**Authors:** Hongyan Huang, Meifang He, Yanbin Zhang, Bo Zhang, Zubiao Niu, You Zheng, Wen Li, Peilin Cui, Xiaoning Wang, Qiang Sun

**Affiliations:** 1 Department of Oncology, Beijing Shijitan Hospital of Capital Medical University, 10 Tieyi Road, Beijing, 100038 China; 2 Laboratory of General Surgery, The First Affiliated Hospital, Sun Yat-Sen University, 58 Zhongshan 2nd Road, Guangzhou, 510080 China; 3 Laboratory of Cell Engineering, Institute of Biotechnology, 20 Dongda Street, Beijing, 100071 China; 4 Department of Gastroenterology, Beijing Tiantan Hospital of Capital Medical University, Beijing, 100070 China

**Keywords:** Gastrointestinal cancer, Prognostic markers, Tumour immunology

**Dear Editor,**

Pancreatic ductal adenocarcinoma (PDAC) has a poor prognosis, with an overall 5-year survival rate of less than 10%. Between 20 and 30% of PDAC cases are resectable at diagnosis; however, patients’ post-operative survival periods vary widely, irrespective of active therapeutic interventions.^1^ Therefore, extensive efforts have been made to identify biomarkers that may identify patients with an improved prognosis. Although profound local immune suppression had been implicated in PDAC progression and poor patient survival, a prognostic marker that can directly and functionally read immune evasion in situ is not yet available.

Cell-in-cell structures (CICs) refer to the presence of one or more cells inside a host cell, which are coordinately driven by a set of core elements including adherens junction, acytomyosin and mechanical ring,^2^ generally leading to inner cell death.^3^ CICs are prevalent in a wide range of human tumors and have different subtypes, including homotypic CICs formed between tumor cells, and heterotypic CICs formed via internalization of immune cells into tumor cells.^3^ Therefore, the heterotypic CICs might serve as a novel mechanism of immune evasion that conceivably promotes cancer progression.^4^ However, the contribution of different CICs, especially heterotypic CICs, to patient prognosis has yet to be established. This study aims to explore the feasibility of using subtyped CICs as a kind of functional biomarker predicting patient survival in PDAC.

In total, 410 specimens from 147 pair-matched cancer and non-malignant tissues and 116 cancer-only tissues, were included in this study. Out of these, 300 specimens, consisting of 147 case-matched non-malignant pancreas controls and 153 cancer tissues (note: only 125 cancer tissues were included into the final analysis due to missing information), were plotted on TMA stained by “EML” multiplex method^5^ (Supplementary Fig. S1) and used as the discovery cohort. The additional 110 validation specimens were collected from the First Affiliated Hospital of Sun Yat-sen University, and, detected by immunohistochemistry staining (Supplementary Fig. S2). The clinical characteristics of the 235 patients included in the final analysis were generally comparable between the discovery and validation cohorts (Supplementary Table S1 and Supplementary Fig. S3).

The CICs in the discovery cohort were minimally detected in non-malignant tissues (15/147) but were prevalent in cancer tissues (97/125) (*p* < 0.0001) (Fig. S1a–e). Four CIC subtypes were identified (Fig. 1a): tumor cells inside tumor cells (TiT), lymphocytes (CD45^+^) inside tumor cells (CDs^−^) (LiT), tumor cells inside macrophages (TiM), and macrophages inside tumor cells (MiT). The subtype of TiT constituted the majority (~70%) of overall CICs (oCICs) (Fig. 1b). Identity analysis indicated that tumor cells were the major engulfer (96.4%) over macrophages (3.6%) (Fig. 1c), and that both tumor cells (73.4%) and immune cells (7.3% CD45^+^ and 19.3% CD68^+^) could be internalized as inner cells (Fig. 1d). A similar CIC profile was identified in the validation cohort (Fig. 1b–d).

**Fig. 1:**
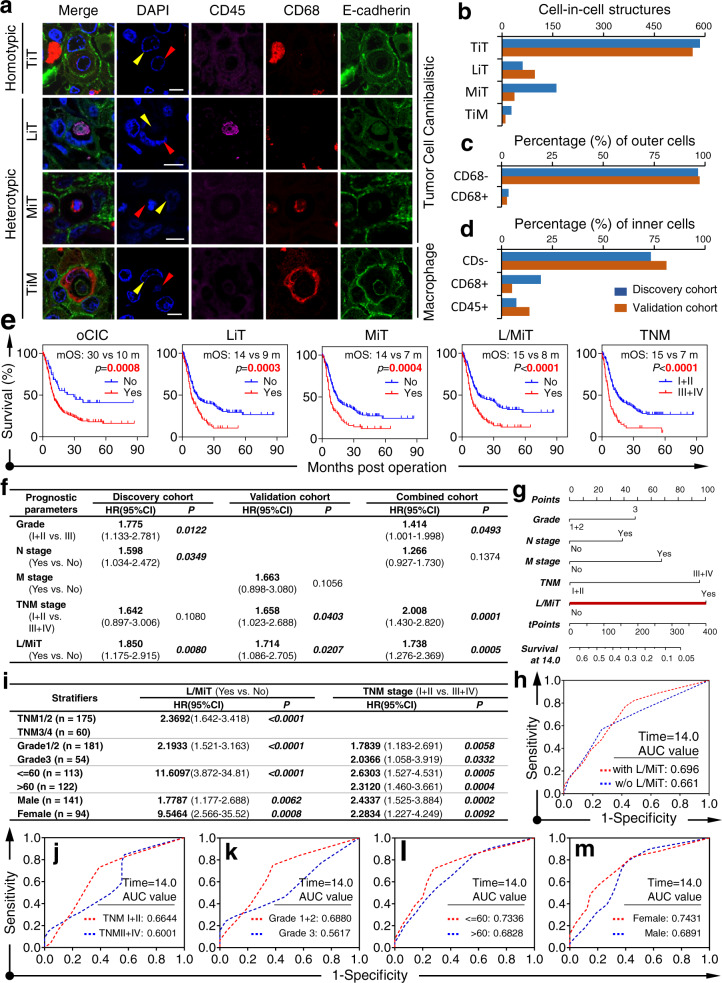
Subtyped CIC structures are associated with the prognosis of patients with resectable PDAC. **a** Representative images for the 4 CIC subtypes identified in human PDAC tissues. TiT: tumor cell in tumor cell; LiT: leukocyte in tumor cell; MiT: macrophage in tumor cell; TiM: tumor cell in macrophage; scale bars: 5 μm. **b**–**d** Profiles of CIC subtypes depicted for all CIC counts (**b**), for outer cell identities (**c**), and for inner cell identities (**d**) in both discovery and validation cohorts. CD68−: negative in CD68. CD68+: positive in CD68. CDs−: negative in both CD68 and CD45. **e** Kaplan–Meier plotting of overall survival curves for indicated variables in combined cohorts of patients, and CIC structures are associated with overall survival of PDAC patients. **f** Multivariate analysis consistently identified L/MiT, the heterotypic CIC subtype, as an independent prognostic factor for PDAC patients across discovery cohort, validation cohort and combined cohort as well. **g** Nomogram analysis with 5 independent prognostic factors (histological grade, N, M, and TNM stage, and L/MiT) identified L/MiT as the prominent prognostic contributor, as indicated by the bar length, at the time point of 14 month-survival in the combined cohorts of patients. tpoints: total points. **h** The AUC calculation of the prediction performance in the combined cohorts of patients in the presence or absence (w/o) of L/MiT. The inclusion of L/MiT increases survival prediction performance. **i** Multivariate analysis of prognostic values of L/MiT and TNM staging stratified by TNM stage, grade, age and sex, respectively, in combined cohort of PDAC patients. L/MiT preferentially impacts younger female patients of early resectable PDAC. **j–m** AUC analysis in combined cohort of patients stratified by TNM stage (I + II vs. III + IV) (**j**), histological grade (1 + 2 vs. 3) (**k**), age (≤ 60 vs. >60) (**l**), and sex (female vs male) (**m**). L/MiT plays dominant role in young female patients with resectable PDAC

In the discovery cohort, homotypic TiT was more frequently detected in tissues of older patients (45/70 for <60 years old vs. 45/55 for ≥60 years old, *p* = 0.03); however, this was not confirmed in the validation dataset. Heterotypic MiT and L/MiT (for LiT plus MiT) tended to be more frequently present in tumor tissues of a late TNM stage (35/109 vs. 11/16, *p* = 0.005) and low tumor differentiation (37/85 vs. 25/40, *p* = 0.048), respectively, which were confirmed in the validation cohort. When combining the discovery and validation datasets, the presence of homotypic TiT also demonstrated a significant association with late TNM stage (*p* = 0.0032) (Supplementary Table S2a–c). These results are consistent with the notion that later stage tumor cells more frequently engage in cannibalistic activities.

Univariate analysis revealed that tumor grade, TNM stage, lymph node (LN) invasion, and distant metastasis were significantly associated with postoperative survival in all 3 cohorts. Of these traditional variables, TNM stage was likely, and expectedly, the most consistent and representative survival classifier across the 3 cohorts. Notably, the presence of oCICs, LiT, MiT and L/MiT was also associated with a shorter OS across the 3 cohorts. The adverse prognostic role of heterotypic CICs may have been specific for CICs of the tumor cell engulfer (L/MiT) as the presence of TiM, the heterotypic CIC subtype of macrophage engulfer, did not significantly impact patient survival (Fig. 1e and Supplementary Fig. S4, Supplementary Table S3–4).

For multivariate survival analysis, we included all of the variables identified in univariate analysis (grade, TNM stage, LN invasion, distant metastasis, oCIC, TiT, LiM, MiT, and L/MiT) into the Cox proportional hazards model. Unexpectedly, TNM stage was not an independent prognostic factor (HR = 1.642, 95%CI: 0.897–3.006, *p* = 0.108) in the discovery cohort, whereas L/MiT was the strongest risk factor for a poor prognosis, with a death hazard ratio of 1.850 (95% CI: 1.175–2.915, *p* = 0.008). And the heterotypic L/MiT was the only variable that was consistently identified as an independent prognostic factor across 3 cohorts of patients (Fig. 1f), suggesting that L/MiT could be a dominant contributor to a poor prognosis.

To directly evaluate L/MiT’s contribution to prognosis, we constructed a nomogram that incorporated all 5 independent prognostic factors identified, each of which was assigned a score on a point scale based on its predictive power from the multivariate analysis. As shown in Fig. 1g, h, despite the fluctuations in the performance of the traditional variables across 3 cohorts, L/MiT consistently dominated over all the traditional factors in predicting patient survival, and incorporating L/MiT improved the prediction performance at the time point of 14.0 months. We also calculated the AUC values at earlier survival time points, which reported similar results as shown in Supplementary Table S5.

To further analyze whether L/MiT preferentially affects a specified portion of patients, we stratified the combined cohort of patients by TNM stage or histological grade for multivariate analysis. Traditional variables identified in univariate analysis (grade, N, M, and TNM stage) except for the stratifiers, and all types of CICs, were examined by Cox proportional hazards model. As shown in Supplementary Table S6–7 and Fig. 1i (first line), L/MiT was the only prognostic factor that could independently predict postoperative OS, specifically in patients of an early TNM stage. The selectivity of L/MiT was also applied to patients stratified by grade, age and sex, but not tumor site, N stage, or M stage (Supplementary Table S12–14), where L/MiT is a highly selective predictor of a poor outcome in young, female and low grade patients while TNM stage demonstrated little patient preference (Fig. 1i and Supplementary Table S8–11). The nomogram construction and AUC analysis confirmed that at 14 months, L/MiT was a selective prognostic classifier that predicts decreased survival in young female patients with resectable PDAC (Fig. 1j–m and Supplementary Fig. S6).

In summary, this study reported the first subtype-based CIC profiling in human PDAC, and identified oCICs and its heterotypic subtypes (LiT, TiM, and L/MiT) as valuable prognostic markers in predicting patient survival in a specified group. L/MiT was identified as a potent adverse prognostic marker impacting young female patients with early-stage PDAC. Our work also supports functional pathology with CIC profiling as a novel input for traditional pathology. Despite these implications, our study presents several limitations which were described detailly in the Discussion section of the Supplemental files.

## Supplementary information

supplementary files
