# Supplementary material for: Identification and validation of heterotypic cell-in-cell structure as an adverse prognostic predictor for young patients of resectable pancreatic ductal adenocarcinoma
Source: Signal Transduct Target Ther. 2020 Oct 20;5:246. doi: 10.1038/s41392-020-00346-w (PMC7576137; doi:10.1038/s41392-020-00346-w)
Supplement: Supplementary file 1 — supplementary files [file 41392_2020_346_MOESM1_ESM.pdf]

Supplementary Materials for

Identification and validation of heterotypic cell-in-cell structure as an adverse prognostic predictor for young patients of resectable pancreatic ductal adenocarcinoma

Hongyan Huang[1*]†, Meifang He[2]†, Yanbin Zhang[1]†, Bo Zhang[1, 3]†, Zubiao Niu[3], You Zheng, [3], Wen Li[2], Peilin Cui[4*], Xiaoning Wang[3*], Qiang Sun[3*]

[1]*Department of Oncology, Beijing Shijitan Hospital of Capital Medical University, 10 Tieyi Road, Beijing 100038, China.*

[2]*Laboratory of General Surgery, The First Affiliated Hospital, Sun Yat-Sen University, 58 Zhongshan 2nd Road, Guangzhou 510080, China.*

[3]*Laboratory of Cell Engineering, Institute of Biotechnology, 20 Dongda Street, Beijing 100071, China.*

[4]*Department of Gastroenterology, Beijing Tiantan Hospital of Capital Medical University, Beijing, 100070, China.*

†These authors contributed equally to this work.

Correspondence to:
hhongy1999@126.com
cuipeilin@bjtth.org
xnwang88@163.com
sunq@bmi.ac.cn

This PDF file includes:

Extended Discussion
Materials and Methods
Figures. S1 to S6
Tables S1 to S17
TRIPOD checklist

Extended Discussion

Overall, our data support the hypothesis that subtyped CICs are promising prognostic markers for human PDAC, and that the presence of CICs is generally associated with a shorter survival time. Meanwhile, the prognostic performance of oCICs was profoundly affected by its subtype composition with the heterotypic CIC being a predominant factor. Furthermore, the active interactions between different CIC subtypes in patient prognosis were consistent with their intrinsically different biological functions. First, the presence of MiT or LiT may indicate that there is an even more malignant phenotype of tumor cell compared to TiT, as they (MiT and LiT) render tumor cells to kill macrophages, T cells, and/or natural killer cells that are designed to kill tumor cells, resulting in a form of immune evasion [1]. Consistent with this notion, the presence of either LiT, MiT, or L/MiT was significantly associated with shorter patient OS, which also reinforces the concept that compromised immunity drives PDAC progression [2]. Second, the presence of TiM may indicate the occurrence of immune activation, whereby tumor cells are eliminated by macrophage phagocytosis which has been shown to be a potential tumor therapy [3,4]. In agreement with this notion, a 65-year-old male patient, whose tumor tissue had a high level of TiM (12/core), survived for as long as 79 months as of his last visit, despite being diagnosed as histological grade 3. Our findings may also help to explain the unexpected protective role of CICs in PDAC metastasis, as reported in a study by Cano *et al.* [5] in which $CD68^+$ CICs were identified but were not quantitatively subtyped. On these grounds, we can speculate that perhaps a considerable presence of TiM actively prevents metastasis. Intriguingly, TiT by entosis, a non-apoptotic cell death mechanism [6], was recently shown to be a mechanism of cell competition which promotes selection of malignant clones [7,8]. The data here therefore suggests that a similar competitive mechanism may work in heterotypic CICs, resulting in tumors evolving to become more immune-resistant [8], and, as such, further functional validation is warranted.

Marker selection is an important part of CIC subtyping. Based on our preliminary study, E-cadherin, CD68, and CD45 are ideal for subtyping PDAC. This study, in line with previous research [9], found that, in comparison with adjacent non-cancerous tissues, downregulation of E-cadherin expression was common in the PDAC tissues. However, only 3 tissues saw a complete loss of E-cadherin expression (Fig. S1f). In these cases, the cell morphology and CICs were identified by overexposure of background fluorescence assisted by H&E staining. Meanwhile, although CD163 was another accepted marker for

macrophage, it was not a good marker for labeling macrophages participating in CIC formation [10]. Instead, CD68 turned out to work well as an identifier of CICs in breast cancer [11-13], esophageal cancer (unpublished data), and pancreatic cancer as illustrated in this study.

An interesting finding of this work is that heterotypic L/MiT preferentially impacts certain groups of patients, specifically young women with early-stage PDAC (TNM I+II, or grade 1+2) (Table 2 and S6-9). In short, the presence of L/MiT in resectable PDAC tissues could independently predict poor outcomes for young female patients, whereas the survival of those without L/MiT was substantially longer. Though the underlying mechanisms warrant further investigation, this selective impact may reflect a dynamic role of a specified mechanism in different contexts. It is widely accepted that the development and progression of cancer is a net outcome of balance between driver and blocker factors [14]. Each factor may dominate cancer progression in a defined context. We assumed that young and/or female PDACs were primarily promoted by the factors of active drivers, where simultaneous loss of blockers, such as immune surveillance by killing immune cells via L/MiT, would significantly potentiate cancer progression and predict poor prognosis. Therefore, our finding is not only informative and helpful for clinical practice but also provocative for further mechanistic investigations. It is conceivable that the formation of heterotypic L/MiT may surrogate the occurrence of specific oncogenic mutations, which, despite being invisible to traditional pathology in resectable PDAC, confer cancer cells the ability to cannibalize immune cells. Hence, exploring the molecular mechanisms underlying heterotypic CIC formation may help identify novel therapeutic targets for resectable PDAC with L/MiT and may benefit patient survival.

One of the most important implications of this work is the application of CICs as a functional index for patient diagnosis and prognosis. Current histological diagnosis largely depends on traditional pathology or molecular pathology, which generally produce information focusing on individual cells or molecules. Because tumors are quite heterogeneous, both in terms of morphology and genetics [15-17], multiple histological parameters are generally required for a relative improvement in tumor malignancy and patient prognosis predictions. Therefore, simple functional parameters that could readout tumor malignancy would be favored by clinicians. CICs arise from active cell-cell interactions between different types of cells within heterogeneous tumor microenvironments [10,18]. Formation of CICs

generally leads to different functional outcomes of inner and outer cells, which can promote the growth of the outer cells while killing the inner cells [19]. During CIC formation, the identities of inner or outer cells are genetically regulated [12], and oncogenic mutations such as *Kras* V12 are able to confer tumor cells outer/winner identity by inhibiting actomyosin contraction [7] while genetic inactivation of tumor suppressor CDKN2a or activation of p53 signaling leads cells to be internalized as inner/loser [12,20]. Thus, CIC is an ideal functional candidate to identify complex intercellular interactions and complicated intracellular signaling crosstalk. Consistent with this notion, our data in this work demonstrated that both oCICs and subtyped CICs (TiT, MiT, L/MiT) were able to predict patient prognoses with a performance comparable or even superior to traditional parameters such as TNM staging. Moreover, CICs were found to be a useful diagnostic indicator to differentiate benignity from malignancy in urothelial carcinoma, malignant mesothelioma, and effusion/urine cytology [21-25]. And a high number of CICs were identified as an adverse prognostic factor for overall survival of patients with head and neck cancers [26], while in early breast cancer, CICs were found capable of selectively impacting patient survival in different categories and significantly contributing to the prediction of patient outcomes [11]. Accordingly, we propose CIC profiling as a promising method to assist with tumor diagnosis and patient prognosis. It may constitute an essential part of an emerging functional pathology that will improve the performance of both traditional and molecular pathology.

According to our findings above, PDAC patients with CIC-positive samples may be at a higher risk of succumbing to poor survival even though they are pathologically at a low histological grade and early TNM stage. For such cases, active treatments and more frequent follow-up should be adopted. It should be noted that our data suggests that L/MiT preferentially impacted young and female patients, particularly those at early-stage, but the predictive values of other CICs subtypes may not be underscored across the entire patient cohort considering the limitations of this study, which is discussed below. Therefore, we recommend that CIC profiling should be performed post-operatively for all PDAC patients together with traditional pathology by using the paraffin-imbedded tumor sections. The consequences of the study reported here, which could be extended to other cancer types, are two-fold, namely: a) the study provides a readout to assess immune evasion and predict prognosis in PDAC; b) the study also supports the notion that the presence of L/MiT associates with cancer progression and shorter survival in cancer patients.

Despite these implications, the impact of the present study was limited by several factors. First, the retrospective nature of this study needs further validation through a prospective study. Second, although this is currently the largest subtype-based CIC profiling of human cancers, the tissue sample size should be expanded in future studies for further confirmation. Third, since commercial TMA was used to explore the prognostic value of CICs in this study, some information, such as neoadjuvant chemotherapy, and subsequent treatments following surgery and date of first relapse, were not available; this prevents us from evaluating the effects various treatments have on both patient survival and disease-free survival (DFS). Also, the approach/marker reported in this study is currently operator-dependent and the characterization of tumors with immunofluorescence may generate technical issues, for which the artificial intelligence-based automated image analysis might be a solution. Moreover, a head to head comparison with other prognostic markers might be needed for the potential clinical application of this approach/marker. In addition, though tumor size is a known prognostic factor for PDACs, it failed to show significant power to discriminate patient survival in this study (Fig. S15-S17), which probably due to limited cohort size. Study with more PDAC patients would be helpful to address this issue. These considerations warrant further investigation in the future.

In summary, this study reported the first subtype-based CIC profiling in human PDAC, and identified oCICs and its heterotypic subtypes (LiT, TiM, and L/MiT) as valuable prognostic markers in predicting patient survival in a specified group. L/MiT was identified as a potent adverse prognostic marker impacting young female patients with early-stage PDAC. Our work also supports functional pathology with CIC profiling as a novel input for traditional pathology, and the promise it holds for improving clinical diagnosis and guiding cancer therapy.

Materials and Methods

Human tumor tissue microarray and PDAC tissue

Two human tumor tissue microarrays (TMA) with paired samples of resectable cancer and non-malignant pancreas tissues from 153 PDAC patients were purchased from Shanghai Outdo Biotech Co. Ltd (HPan-Ade180Sur-01 and HPan-Ade120Sur-01). The Outdo Biotech is a leading company in human/animal tissue microarrays (TMA) and "clinical-type" gene chips (CTGCs) in China. All tissues were collected under the highest ethical standards with the donors being fully informed and their

consent being obtained. The samples were collected from patients between 2004 and 2008 with follow-up until 30 June 2014 and stored and transported at -80℃. The cases were routinely followed up by professional doctors. The TMA slide was prepared from formalin-fixed, paraffin-embedded cancer and paired non-malignant tissue. In total, there were 300 cores on 2 slides, including 153 cases of pancreatic cancer tissues and 147 cases of non-malignant tissues (not plotted for 6 patients). The diameter of each core was 1.5 mm (1.76 mm^2/core). For validation, tissue sections were collected from 110 resectable PDAC patients between 2003 and 2011 with follow-up until November 2012. All patients had received surgery at the Department of Hepatobiliary Surgery, the First Affiliated Hospital of Sun Yat-sen University (Guangzhou, China), with the institutional research ethics committee reviewing and approving the research. All of the patients received ultrasound and computed tomography scans prior to surgery and none received systemic chemotherapy or radiotherapy preoperatively. All specimens were diagnosed by pathological examination after surgery.

TMA and tissue specimen staining, and antibodies

The “EML method”, a multiplexing method based on the technique of tyramide signal amplification (TSA) [10], was employed to subtype CICs. Through this method, tissues were simultaneously stained with antibodies against E-cadherin for epithelial cancer cells, CD45 for leukocytes, and CD68 for macrophages. Slides were routinely de-paraffinized with the xylene-ethanol method and baked at 65°C for 1.5 hours. Antigen retrieval was performed in citrate acid buffer by microwaving for 15 minutes after boiling, followed by 1 hour blocking in 5% bovine serum albumin (BSA) made in Tris-buffered saline (TBS). Samples were first stained with anti-CD45 antibody (mouse mAb from Boster, BM0091) at a dilution of 1:400 using Opal Multiplex tissue staining kit (Perkin Elmer, NEL791001KT) according to the manufacturer’s standard protocol. CD45 molecules were subsequently labeled with Cyanine 5 fluorophore. The slides were then incubated with mixed antibodies for E-Cadherin (1:200, mouse mAb from BD Biosciences, 610181) and CD68 (1:200, rabbit pAb from Proteintech, 25747–1-AP), followed by Alexa Fluor 568 secondary anti-rabbit antibody (Invitrogen, A11036) and Alexa Fluor 488 anti-mouse antibody (Invitrogen, A11029). Samples were also labeled with single fluorophore to acquire spectral signatures. All slides were counterstained with DAPI to show nuclei, before being mounted with Antifade reagent (Invitrogen, Carlsbad, CA, USA) and cover slips, and then sealed with clear nail polish. For validation, tissue sections were stained by hematoxylin and eosin (H&E) and

immunohistochemistry (IHC) with each of the antibodies indicated above, following the protocol provided by Cell Signaling Technology (https://www.cellsignal.com/contents/resources-protocols/immunohistochemistry-protocol-paraffin-for-signalstain-boost-detection-reagent/ihc-paraffin-signalstain).

Multispectral imaging and analysis

Multispectral images were taken with TMA modules of Vectra® Automated Imaging System (Perkin Elmer) by a 20x objective lens (Fig. S1). A nuance system (Perkin Elmer) was used to build libraries of each spectrum (DAPI, 488, 568, and Cy5-650) and unmix multispectral images with high contrast and accuracy (Fig. S1). inForm automated image analysis software package (Perkin Elmer) was used for batch analysis of multispectral images based on specified algorithms.

CIC profiling and quantification

Cellular structures were scored as CICs where one or more cells morphologically were fully enclosed within another cell with a crescent nucleus. As CICs can result in inner cell death, we scored all structures displaying CIC morphology irrespective of whether inner cells were dead or live. Cell boundaries were identified by E-cadherin, which labels cell membranes, and/or CD68, which labels cell bodies. CIC subtypes were defined based on the types of cells involved: TiT for E-cadherin^{+} cells inside E-cadherin^{+} cells; TiM for E-cadherin^{+} cells inside CD68^{+} cells, MiT for CD68^{+} cells inside E-cadherin^{+} cells, LiT for CD45^{+} cells inside E-cadherin^{+} cells. For efficient quantification of CICs in TMA, the whole area of each core was first screened in a composite image of 4 fluorescent channels and then confirmed in unmixed channels. For quantification in validation specimens, images from 10 random fields of 400x magnification were analyzed for each sample, subtyped CICs were counted based on IHC staining with reference to H&E staining (Fig. S2). Double-blind reviews were performed for all the CIC quantifications.

Statistical analysis

Statistical analysis was performed using the SPSS 20.0 (IBM Corp., NY, USA) and EmpowerStats (http://www.empowerstats.com/) software systems, which wraps R software. Study data were collected on standard forms and checked for completeness. All data were described using median (min–max) for continuous variables like follow-up times, while frequencies (percent) were used for categorical

variables. Overall survival (OS) was defined as time from the date of surgery to death or to the most recent contact or visit. The follow up times were provided along with TMA slide for the discovery cohort, where the longest survival time is 87 months (Fig S4). The follow up times were obtained from patient data sheet for the validation cohort, where the longest survival time is 58 months (Fig S4). Survival times were analyzed by the Kaplan–Meier method, and the differences in survival times were compared by the log-rank test. Univariate and multivariate survival analyses were performed using the Cox proportional-hazard models, and hazard ratios (HRs) (95% confidence interval) were calculated. The association between clinicopathological factors and the number of CICs was analyzed using the Chi-square test or Fisher's exact test. The nomogram was formulated based on the results of multivariate logistic regression analysis by Regression Modeling Strategies, which proportionally converts each regression coefficient in multivariate logistic regression to a 0-to-100-point scale as described elsewhere [27]. The area under the curve (AUC) calculation was performed and graphed with EmpowerStats software. For all analyses, a two-sided p value of less than 0.05 was considered statistically significant.

Data Availability

All data and materials are available to the researchers once published.

References

1 Wang, S. *et al.* Rapid reuptake of granzyme B leads to emperitosis: an apoptotic cell-in-cell death of immune killer cells inside tumor cells. *Cell death & disease* **4**, e856, doi:10.1038/cddis.2013.352 (2013).

2 Balachandran, V. P., Beatty, G. L. & Dougan, S. K. Broadening the Impact of Immunotherapy to Pancreatic Cancer: Challenges and Opportunities. *Gastroenterology* **156**, 2056-2072, doi:10.1053/j.gastro.2018.12.038 (2019).

3 Chao, M. P., Weissman, I. L. & Majeti, R. The CD47-SIRPalpha pathway in cancer immune evasion and potential therapeutic implications. *Current opinion in immunology* **24**, 225-232, doi:10.1016/j.coi.2012.01.010 (2012).

4 Feng, M. *et al.* Macrophages eat cancer cells using their own calreticulin as a guide: roles of TLR and Btk. *Proc Natl Acad Sci U S A* **112**, 2145-2150, doi:10.1073/pnas.1424907112 (2015).

5 Cano, C. E. *et al.* Homotypic cell cannibalism, a cell-death process regulated by the nuclear protein 1, opposes to metastasis in pancreatic cancer. *EMBO Mol Med* **4**, 964-979, doi:10.1002/emmm.201201255 (2012).

6 Overholtzer, M. *et al.* A nonapoptotic cell death process, entosis, that occurs by cell-in-cell invasion. *Cell* **131**, 966-979, doi:10.1016/j.cell.2007.10.040 (2007).

7 Sun, Q. *et al.* Competition between human cells by entosis. *Cell Res* **24**, 1299-1310, doi:10.1038/cr.2014.138 (2014).

8 Sun, Q., Huang, H. & Overholtzer, M. Cell-in-cell structures are involved in the competition between cells in human tumors. *Mol Cell Oncol* **2**, e1002707, doi:10.1080/23723556.2014.1002707 (2015).

9 Hong, S. M. *et al.* Loss of E-cadherin expression and outcome among patients with resectable pancreatic adenocarcinomas. *Mod Pathol* **24**, 1237-1247, doi:10.1038/modpathol.2011.74 (2011).

10 Huang, H. *et al.* Detecting cell-in-cell structures in human tumor samples by E-cadherin/CD68/CD45 triple staining. *Oncotarget* **6**, 20278-20287 (2015).

11 Zhang, X. *et al.* Subtype-Based Prognostic Analysis of Cell-in-Cell Structures in Early Breast Cancer. *Front Oncol* **9**, 1-12, doi:10.3389/fonc.2019.00895 (2019).

12 Liang, J. *et al.* CDKN2A inhibits formation of homotypic cell-in-cell structures. *Oncogenesis* **7**, 1-8, doi:10.1038/s41389-018-0056-4 (2018).

13 Ruan, B. *et al.* High Frequency of Cell-in-Cell Formation in Heterogeneous Human Breast Cancer Tissue in a Patient With Poor Prognosis: A Case Report and Literature Review. *Front Oncol* **9**, 1-6, doi:10.3389/fonc.2019.01444 (2019).

14 Hanahan, D. & Weinberg, R. A. Hallmarks of cancer: the next generation. *Cell* **144**, 646-674, doi:10.1016/j.cell.2011.02.013 (2011).

15 Campbell, P. J. *et al.* The patterns and dynamics of genomic instability in metastatic pancreatic cancer. *Nature* **467**, 1109-1113, doi:10.1038/nature09460 (2010).

16 Waclaw, B. *et al.* A spatial model predicts that dispersal and cell turnover limit intratumour heterogeneity. *Nature* **525**, 261-264, doi:10.1038/nature14971 (2015).

17 Roberts, N. J. *et al.* Whole Genome Sequencing Defines the Genetic Heterogeneity of Familial Pancreatic Cancer. *Cancer discovery* **6**, 166-175, doi:10.1158/2159-8290.CD-15-0402 (2016).

18 Huang, H., Chen, Z. & Sun, Q. Mammalian Cell Competitions, Cell-in-Cell Phenomena and Their Biomedical Implications. *Current molecular medicine* **15**, 852-860, doi:10.2174/1566524015666151026101101 (2015).

19 Fais, S. & Overholtzer, M. Cell-in-cell phenomena in cancer. *Nat Rev Cancer* **18**, 758-766, doi:10.1038/s41568-018-0073-9 (2018).

20 Liang, J. *et al.* Counteracting Genome Instability by p53-dependent Mintosis. *bioRxiv*, doi:10.1101/2020.01.16.908954 (2020).

21 Washiya, K. *et al.* Cytologic difference between benignity and malignancy in suspicious cases employing urine cytodiagnosis using a liquid-based method. *Anal Quant Cytol Histol* **33**, 169-174 (2011).

22 Bansal, C., Tiwari, V., Singh, U., Srivastava, A. & Misra, J. Cell Cannibalism: A cytological study in effusion samples. *J Cytol* **28**, 57-60, doi:10.4103/0970-9371.80736 (2011).

23 Kimura, N., Dota, K., Araya, Y., Ishidate, T. & Ishizaka, M. Scoring system for differential diagnosis of malignant mesothelioma and reactive mesothelial cells on cytology specimens. *Diagn Cytopathol* **37**, 885-890, doi:10.1002/dc.21128 (2009).

24 Gupta, K. & Dey, P. Cell cannibalism: diagnostic marker of malignancy. *Diagn Cytopathol* **28**, 86-87, doi:10.1002/dc.10234 (2003).

25 Matsumoto, S. *et al.* Morphology of 9p21 homozygous deletion-positive pleural mesothelioma cells analyzed using fluorescence in situ hybridization and virtual microscope system in effusion cytology. *Cancer Cytopathol* **121**, 415-422, doi:10.1002/cncy.21269 (2013).

26 Schwegler, M. *et al.* Prognostic Value of Homotypic Cell Internalization by Nonprofessional Phagocytic Cancer Cells. *BioMed research international* **2015**, 359392, doi:10.1155/2015/359392 (2015).

27 Steyerberg, E. W. & Vergouwe, Y. Towards better clinical prediction models: seven steps for development and an ABCD for validation. *European heart journal* **35**, 1925-1931, doi:10.1093/eurheartj/ehu207 (2014).

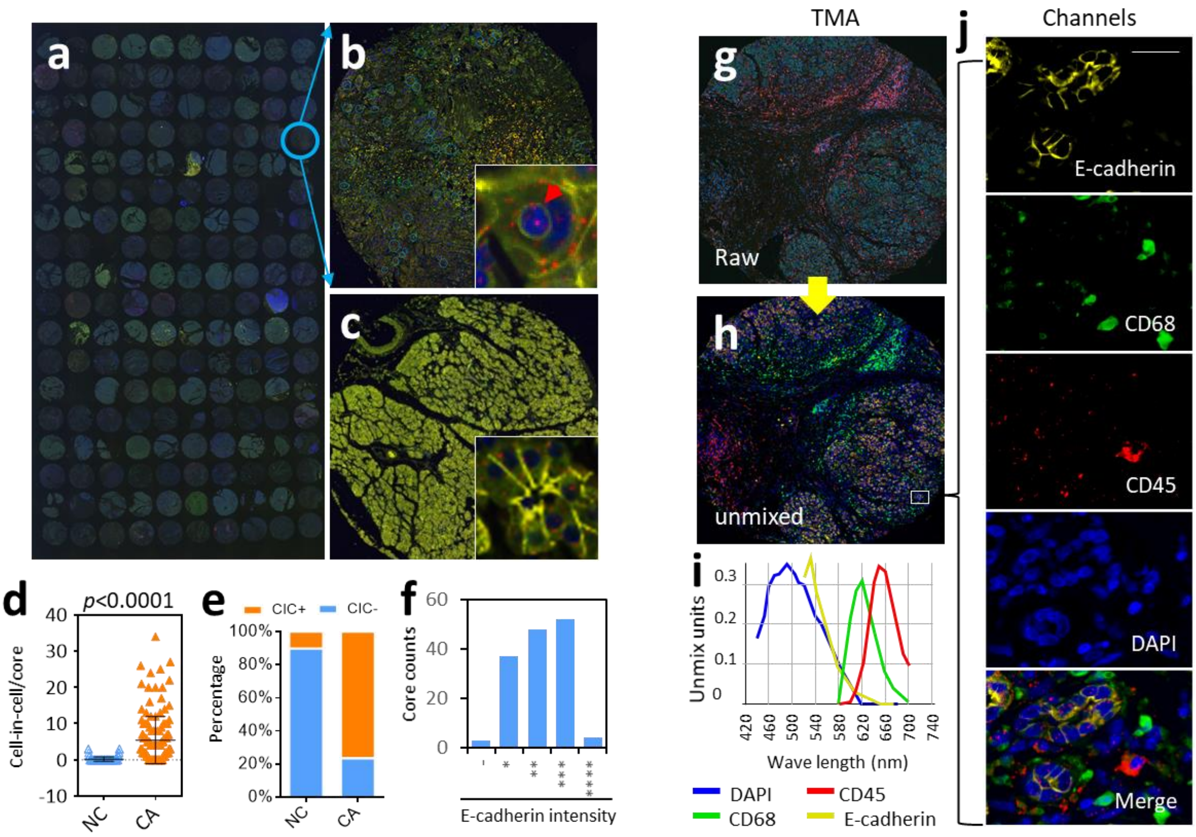

Fig. S1

Detecting CIC structures in tissue microarray (TMA) of human PDAC. (**a-b**) Composite images of whole TMA slide (**a**), single representative cancer tissue core (**b**) and para-cancer tissue core (**c**). Inserted images show one typical CIC structure, or acinar. Arrow indicates inner cell. E-cadherin staining (in yellow) indicates intercellular contact; (**d**) The profiles of all CICs detected are depicted as CIC number per core in cancer and non-cancerous tissues; (**e**) The percentage of CIC+ and CIC- tissues in cancer or para-cancer samples, respectively. (**f**) E–cadherin expression profile in pancreatic cancerous tissue cores. "–" for negative, "****" for strongest expression comparable to the adjacent non-malignant tissues. n=144. (**g**) Unprocessed composite core multiplex stained with E-cadherin, CD68, CD45, and DAPI. (**h**) Unmixed composite core pseudo-colored with yellow for E-cadherin, green for CD68, red for CD45, and blue for DAPI. (**i**) Spectral parameters for image unmixing. Fluorescent signals for different targets were captured based on the spectra indicated. (**J**) Images of single or merged channels displaying the boxed region in (**c**). Scale bar: 20 μm.

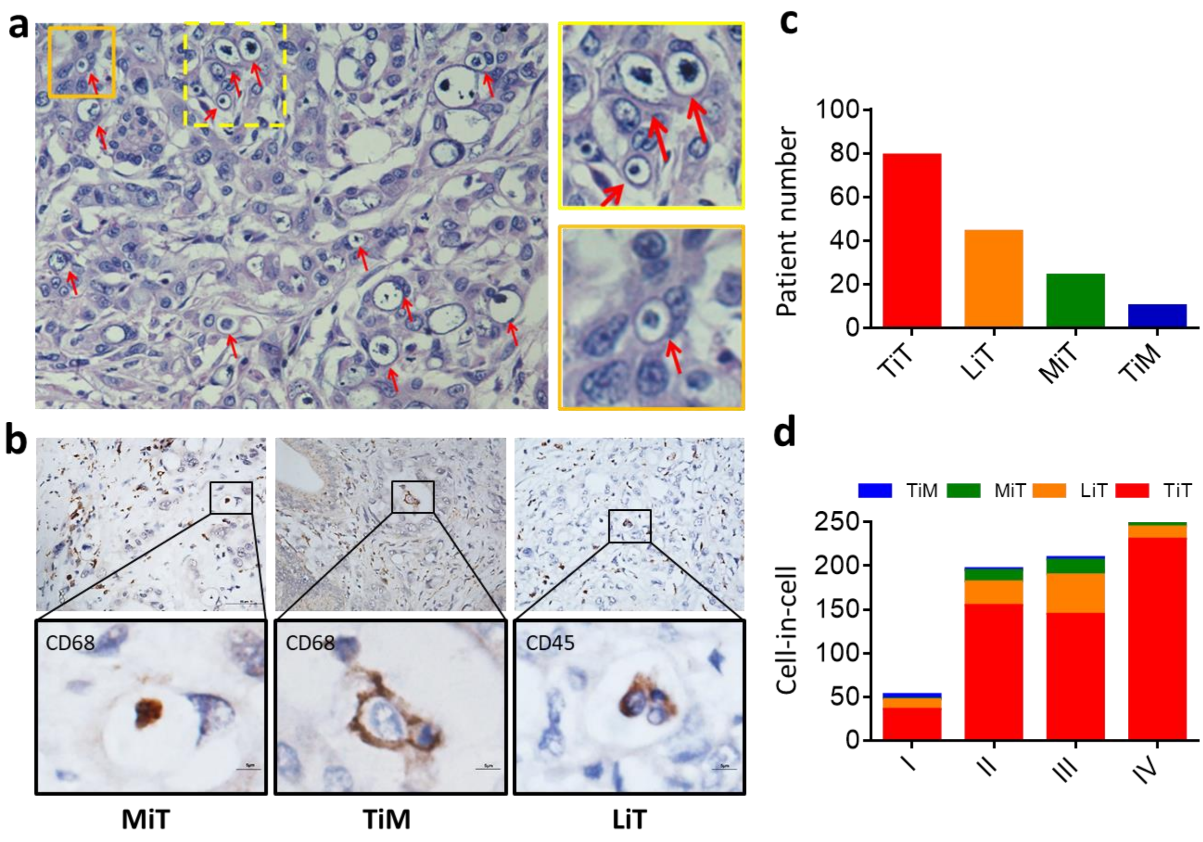

Fig. S2

Detecting CIC structures in human PDAC of validation cohort. (**a**) CIC structures indicated by arrows in PDAC tissues stained by H&E. Boxed regions are zoomed in at the right. (**b**) CIC structures in PDAC tissues stained with antibodies for CD68 or CD45, respectively, by IHC. Boxed regions are zoomed in at the bottom. (**c**) Number of tissues positive in each CIC subtype; (**d**) The compositions of CIC subtypes in different TNM stages. Type or paste caption here. Create a page break and paste in the Figure above the caption.

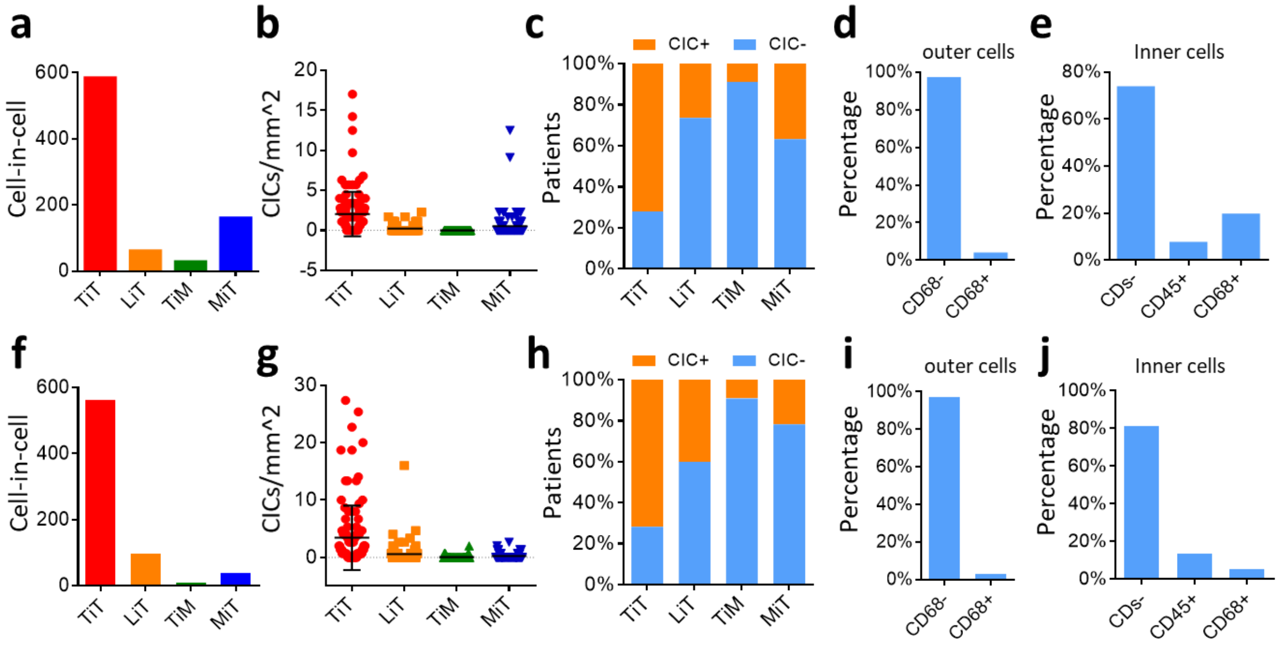

Fig. S3

CIC profiling in discovery (a–e) and validation (f–j) cohorts. Profiles of CICs subtypes depicted for all CIC counts (**a, f**), for CIC counts normalized by core area (**b, g**), for patient percentages (**c, h**), for outer cell identities (**d, i**), and for inner cell identities (**e, j**).

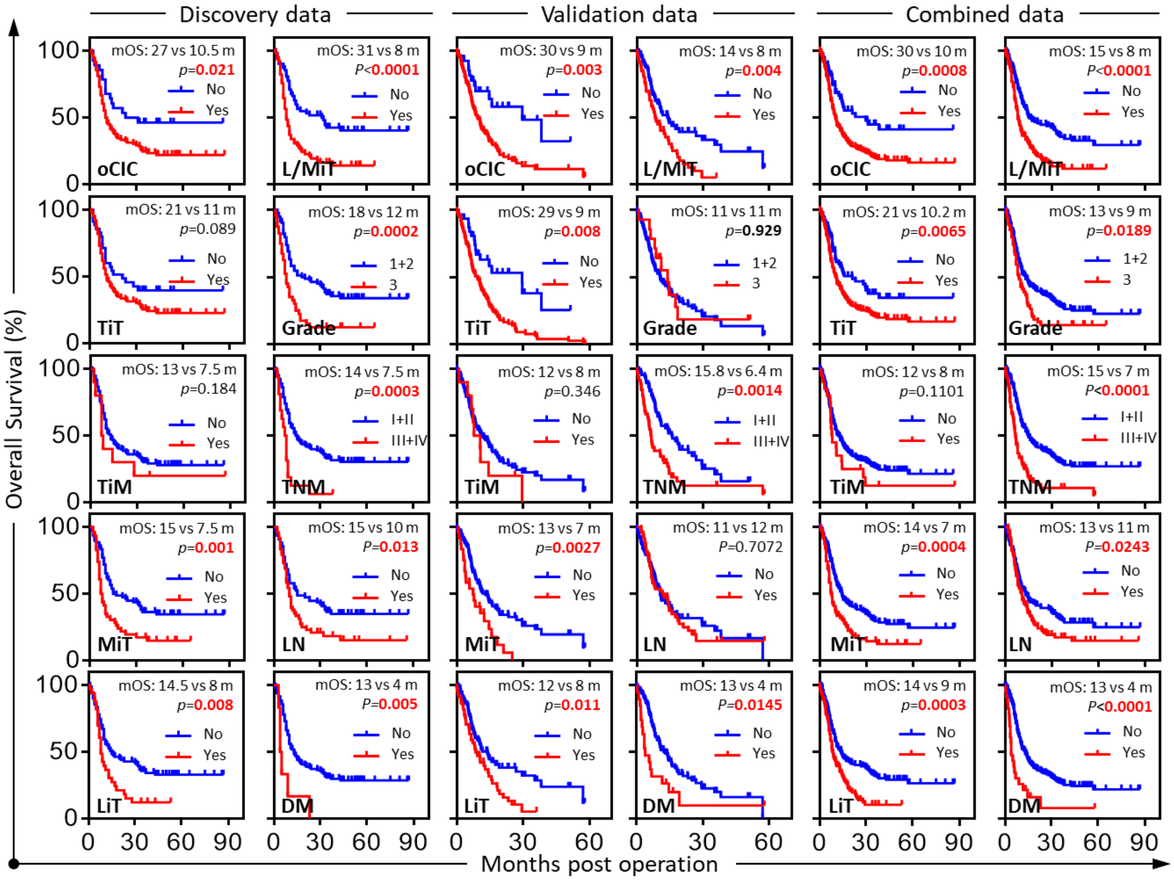

Fig. S4

Kaplan-Meier plotting of overall survival curves for indicated variables across different cohorts of patients. LN for lymph node invasion; DM for distant metastasis.

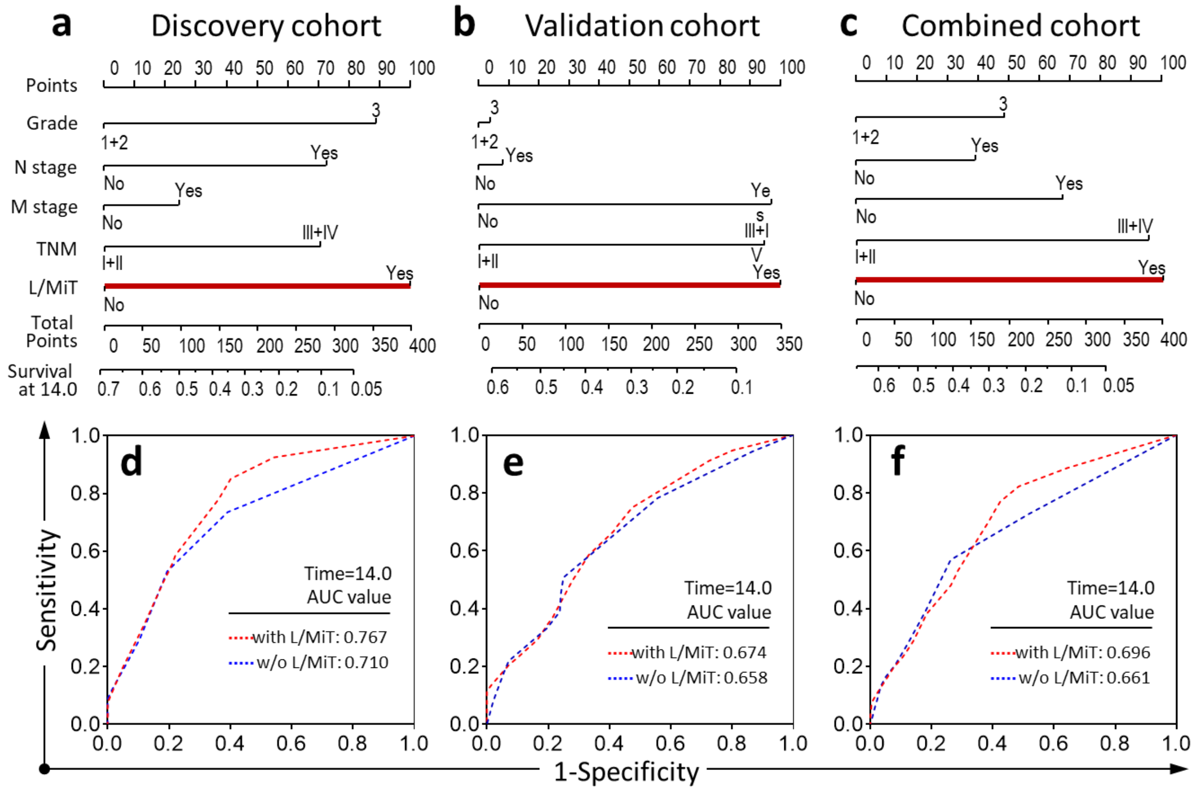

Fig. S5

Contribution of heterotypic L/MiT to survival prediction by nomogram analysis. (**a–c**) Nomogram analysis with 5 independent prognostic factors identified (histological grade, N, M, and TNM stage, and L/MiT). L/MiT stands out as the dominant prognostic contributor across the discovery, validation, and combined cohorts of patients. (**d–f**) The AUC calculation for nomogram analysis of different cohorts of patients in the presence or absence of L/MiT.

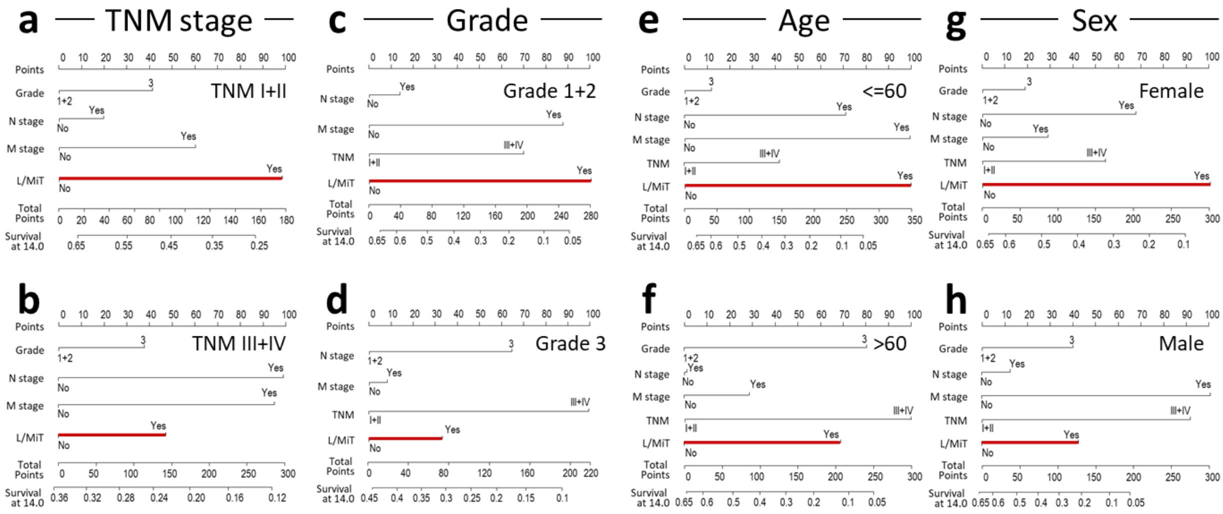

Fig. S6

L/MiT selectively impacts overall survival of certain groups of PDAC patients. Nomogram analysis in combined cohort of patients stratified by TNM stage (I+II vs. III+IV) (**a–b**), histological grade (1+2 vs. 3) (**c–d**), age (≤ 60 vs. > 60) (**e–f**), and sex (female vs male) (**g–h**). L/MiT plays dominant role in young female patients with resectable PDAC.

Table S1 Characteristics for different cohorts of patients

Characteristics	Discovery cohort No (%), n = 125	Validation cohort No (%), n = 110	Entire cohort No (%), n = 235
**Age at diagnosis**			
<60	70 (56.0)	43 (39.1)	113 (48.1)
≥60	55 (44.0)	67 (60.9)	122 (51.9)
**Sex**			
Male	75 (60.0)	66 (60.0)	141 (60.0)
Female	50 (40.0)	44 (40.0)	94 (40.0)
**Site**			
Head	88 (70.4)	81 (73.6)	169 (71.9)
Neck/tail	37 (29.6)	29 (26.4)	66 (28.1)
**Histologic grade**			
I	3 (2.4)	14 (132.7)	17 (7.2)
II	82 (65.6)	63 (57.3)	145 (61.7)
III	40 (32.0)	33 (30.0)	73 (31.1)
**TNM stage**			
I	43 (34.4)	22 (20.0)	65 (27.7)
II	66 (52.8)	44 (40.0)	110 (46.8)
III	10 (8.0)	25 (22.7)	35 (14.9)
IV	6 (4.8)	19 (17.3)	25 (10.6)
**T stage**			
T1	11 (8.8)	11 (10.0)	22 (9.4)
T2	68 (54.4)	19 (17.3)	87 (37.0)
T3/T4	48 (38.4)	80 (72.7)	128 (54.5)
**Lymph node invasion**			
No	76 (60.8)	62 (56.4)	138 (58.7)
Yes	49 (39.2)	48 (43.6)	97 (41.3)
**Distant metastasis**			
M0	119 (95.2)	91 (82.7)	210 (89.4)
M1	6 (4.8)	19 (17.3)	25 (10.6)

Table S2a: Association of CIC subtypes with clinicopathological characteristics for the discovery dataset

Characteristics	n	oCIC (%)		*p*	TiT (%)		*p*	LiT (%)		*p*	MiT (%)		*P*	TiM (%)		*p*	L/MiT (%)		*p*
		=0	≥1		=0	≥1		=0	≥1		=0	≥1		=0	≥1		=0	≥1	
**Age**																			
<60	70	19(27.1)	51(72.9)	0.151	25(35.7)	45(64.3)	***0.030***	52(74.3)	18(25.7)	0.844	45(64.3)	25(35.7)	0.776	65(92.9)	5(7.1)	0.675	38(54.3)	32(45.7)	0.327
≥60	55	9(16.4)	46(83.6)		10(18.2)	45(81.8)		40(72.7)	15(27.3)		34(61.8)	21(38.2)		49(89.1)	6(10.9)		25(45.5)	30(54.5)	
**Gender**																			
Male	75	16(21.3)	59(75.7)	0.726	20(26.7)	55(73.3)	0.684	56(74.7)	19(25.3)	0.740	46(61.3)	29(38.7)	0.596	69(92.0)	6(8.0)	0.949	38(50.7)	37(49.3)	0.942
Female	50	12(24.0)	38(76.0)		15(30.0)	35(70.0)		36(72.0)	14(28.0)		33(66.0)	17(34.0)		45(90.0)	5(10.0)		25(50.0)	25(50.0)	
**Site**																			
Head	88	20(22.7)	68(77.3)	0.892	25(28.4)	63(71.6)	0.875	62(70.5)	26(29.5)	0.219	56(63.6)	32(36.4)	0.876	81(92.0)	7(8.0)	0.866	44(50.0)	44(50.0)	0.890
Neck/tail	37	8(21.6)	29(78.4)		10(27.0)	27(73.0)		30(81.1)	7(18.9)		23(62.2)	14(37.8)		33(89.2)	4(10.8)		19(51.4)	18(48.6)	
**Grade**																			
I+II	85	22(25.9)	63(74.1)	0.173	25(29.4)	60(70.6)	0.608	67(78.8)	18(21.2)	0.053	58(68.2)	27(31.8)	0.089	77(90.6)	8(9.4)	0.989	48(56.5)	37(43.5)	***0.048***
III	40	6(15.0)	34(85.0)		10(25.0)	30(75.0)		25(62.5)	15(37.5)		21(52.5)	19(47.5)		37(92.5)	3(7.5)		15(37.5)	25(62.5)	
**TNM stage**																			
I+II	109	25(22.9)	84(77.1)	0.957	31(28.4)	78(71.6)	1.000	80(73.4)	29(26.6)	1.000	74(67.9)	35(32.1)	***0.005***	100(91.7)	9(8.3)	0.931	58(53.2)	51(46.8)	0.101
III+IV	16	3(18.8)	13(81.3)		4(25.0)	12(75.0)		12(75.0)	4(25.0)		5(31.3)	11(68.8)		14(57.5)	2(12.5)		5(31.3)	11(68.8)	
**T stage**																			
T1+T2	77	17 (22.0)	60 (77.9)	0.9129	22 (28.6)	55 (71.4)	0.8570	52 (67.5)	25 (32.5)	0.0513	47 (61.0)	30 (38.9)	0.5257	70 (90.9)	7 (9.09)	0.8844	35 (45.5)	42 (54.6)	0.1613
T3+T4	48	11 (22.9)	37 (77.1)		13 (27.1)	35 (72.9)		40 (83.3)	8 (16.7)		32 (66.7)	16 (33.3)		44 (91.7)	4 (8.33)		28 (58.3)	20 (41.7)	
**LN metastasis**																			
No	76	17(22.4)	59(77.6)	0.992	21(27.6)	55(72.4)	0.909	56(73.7)	20(26.3)	0.979	50(65.8)	26(34.2)	0.455	70(92.1)	6(7.9)	0.903	39(51.3)	37(48.7)	0.799
Yes	49	11(22.4)	38(77.6)		14(28.6)	35(71.4)		36(73.5)	13(26.5)		29(59.2)	20(40.8)		44(89.8)	5(10.2)		24(49.0)	25(51.0)	
**Distant metastasis**																			
M0	119	27(22.7)	92(77.3)	1.000	33(27.7)	86(72.3)	1.000	87(73.1)	32(26.9)	0.936	78(65.5)	41(34.5)	***0.047***	108(90.8)	11(9.2)	1.000	62(52.1)	57(47.9)	0.202
M1	6	1(16.7)	5(83.3)		2(33.3)	4(66.7)		5(83.3)	1(16.7)		1(16.7)	5(83.3)		6(100.0)	0(0.0)		1(16.7)	5(83.3)	
**Nerve or lymphatic vessel invasion**																			
No	63	17(27.0)	46(73.0)	0.215	19(30.2)	44(69.8)	0.588	43(68.3)	20(31.7)	0.172	39(61.9)	24(38.1)	0.762	56(88.9)	7(11.1)	0.358	30(47.6)	33(52.4)	0.531
Yes	62	11(17.7)	51(82.3)		16(25.8)	46(74.2)		49(79.0)	13(21.0)		40(64.5)	22(35.5)		58(93.5)	4(6.5)		33(53.2)	29(46.8)	

Table S2b: Association of CIC subtypes with clinicopathological characteristics for the validation dataset

Characteristics	n	oCIC (%)		*p*	TiT (%)		*p*	LiT (%)		*p*	MiT (%)		*P*	TiM (%)		*p*	L/MiT (%)		*p*
		=0	≥1		=0	≥1		=0	≥1		=0	≥1		=0	≥1		=0	≥1	
**Age**																			
≤60	43	13 (30.2)	30 (69.8)	0.2669	15 (34.9)	28 (65.1)	0.2107	30 (69.8)	13 (30.2)	0.0939	36 (83.7)	7 (16.3)	0.2598	41 (95.4)	2 (4.6)	0.1944	29 (67.4)	14 (32.6)	0.1147
>60	67	14 (20.9)	53 (79.1)		16 (23.9)	51 (76.1)		36 (53.7)	31 (46.3)		50 (74.6)	17 (25.4)		59 (88.1)	8 (11.9)		35 (52.2)	32 (47.8)	
**Gender**																			
Male	66	20(30.3)	46(69.7)	0.086	23(34.8)	43(65.2)	0.057	38(57.6)	28(42.4)	0.525	50(75.8)	16(24.2)	0.451	59(89.4)	7(10.6)	0.498	37(56.1)	29(43.9)	0.581
Female	44	7(15.9)	37(84.1)		8(18.2)	36(81.8)		28(63.6)	16(36.4)		36(81.8)	8(18.2)		41(93.2)	3(6.8)		27(61.4)	17(38.6)	
**Site**																			
Head	81	21(25.9)	60(74.1)	0.574	24(29.6)	57(70.4)	0.573	47(58.0)	34(42.0)	0.480	62(76.5)	19(23.5)	0.487	72(88.9)	9(11.1)	0.218	46(56.8)	35(43.2)	0.621
Neck/tail	29	6(20.7)	23(79.3)		7(24.1)	22(75.9)		19(65.5)	10(34.5)		24(82.8)	5(17.2)		28(96.6)	1(3.4)		18(62.1)	11(37.9)	
**Grade**																			
I+II	77	27(34.6)	51(65.4)	***<0.0001***	30(39.0)	47(61.0)	***<0.0001***	52(67.5)	25(32.5)	***<0.0001***	63(81.8)	14(18.2)	0.970	73(94.8)	4(5.2)	***0.030***	50(64.9)	27(35.1)	***0.028***
III	33	0(0.0)	33(100.0)		1 (3.0)	32(97.0)		14(32.6)	29(67.4)		23(82.1)	10(17.9)		27(81.8)	6(18.2)		14(42.4)	19(57.6)	
**TNM stage**																			
I+II	66	23(34.8)	43(65.2)	***0.002***	27(70.6)	39(29.4)	***<0.0001***	42(70.6)	24(29.4)	0.340	58(87.9)	8(12.1)	***0.003***	60(90.9)	6(9.1)	1.000	41(62.1)	25(37.9)	0.305
III+IV	44	4(9.1)	40(90.9)		4(45.0)	40(55.0)		24(45.0)	20(55.0)		28(63.6)	16(36.4)		40(90.9)	4(9.1)		23(52.3)	21(47.7)	
**T stage**																			
T1+T2	30	10(33.3)	20(66.7)	0.190	11(36.7)	19(63.3)	0.226	24(80.0)	6(20.0)	***0.009***	27(90.0)	3(10.0)	0.066	28(93.3)	2 (6.7)	0.576	23(76.7)	7(23.3)	***0.016***
T3+T4	80	17(21.3)	63(78.8)		20(25.0)	60(75.0)		42(52.5)	38(47.5)		59(73.8)	21(26.3)		71(89.9)	8(10.1)		41(51.3)	39(48.8)	
**LN metastasis**																			
No	62	17(27.4)	45(72.6)	0.426	22(35.5)	40(64.5)	0.053	40(64.5)	22(35.5)	0.272	52(83.9)	10(16.1)	0.101	56(90.3)	6(9.7)	0.808	38(61.3)	24(38.7)	0.453
Yes	48	10(20.8)	38(79.2)		9(18.8)	39(81.3)		26(54.2)	22(45.8)		34(70.8)	14(29.2)		44(91.7)	4(8.3)		26(54.2)	22(45.8)	
**Liver metastasis**																			
M0	91	26(28.6)	65(71.4)	***0.039***	30(48.4)	32(51.6)	***0.001***	55(60.4)	36(39.6)	0.837	73(82.0)	16(18.0)	0.181	82(90.1)	9(9.9)	0.523	54(59.3)	37(40.7)	0.590
M1	19	1(5.3)	18(94.7)		1 (5.3)	18(94.7)		11(57.9)	8(42.1)		13(68.4)	6(31.6)		18(94.7)	1(5.3)		10(52.6	9(47.4)	

Table S2c: Association of CIC subtypes with clinicopathological characteristics for the combined dataset

Characteristics	n	oCIC (%)		p	TiT (%)		p	LiT (%)		p	MiT (%)		P	TiM (%)		p	L/MiT (%)		p
		=0	≥1		=0	≥1		=0	≥1		=0	≥1		=0	≥1		=0	≥1	
**Age**																			
≤60	113	32 (28.32)	81 (71.68)	0.0868	40 (35.40)	73 (64.60)	***0.0164***	82 (72.57)	31 (27.43)	0.0937	81 (71.68)	32 (28.32)	0.6356	106 (93.81)	7 (6.19)	0.1562	67 (59.29)	46 (40.71)	0.1202
>60	122	23 (18.85)	99 (81.15)		26 (21.31)	96 (78.69)		76 (62.30)	46 (37.70)		84 (68.85)	38 (31.15)		108 (88.52)	14 (11.48)		60 (49.18)	62 (50.82)	
**Gender**																			
Male	141	36 (25.53)	105 (74.47)	0.3454	43 (30.50)	98 (69.50)	0.3138	94 (66.67)	47 (33.33)	0.8205	96 (68.09)	45 (31.91)	0.3824	128 (90.78)	13 (9.22)	0.8519	75 (53.19)	66 (46.81)	0.7485
Female	94	19 (20.21)	75 (79.79)		23 (24.47)	71 (75.53)		64 (68.09)	30 (31.91)		69 (73.40)	25 (26.60)		86 (91.49)	8 (8.51)		52 (55.32)	42 (44.68)	
**Site**																			
Head	169	41 (24.26)	128 (75.74)	0.6199	50 (29.59)	119 (70.41)	0.4127	110 (65.09)	59 (34.91)	0.2622	119 (70.41)	50 (29.59)	0.9140	153 (90.53)	16 (9.47)	0.6478	91 (53.85)	78 (46.15)	0.9230
Neck/tail	66	14 (21.21)	52 (78.79)		16 (24.24)	50 (75.76)		48 (72.73)	18 (27.27)		46 (69.70)	20 (30.30)		61 (92.42)	5 (7.58)		36 (54.55)	30 (45.45)	
**Grade**																			
I+II	181	44 (24.31)	137 (75.69)	0.5485	49 (27.07)	132 (72.93)	0.5269	123 (67.96)	58 (32.04)	0.6660	131 (72.38)	50 (27.62)	0.1844	163 (90.06)	18 (9.94)	0.3211	102 (56.35)	79 (43.65)	0.1931
III	54	11 (20.37)	43 (79.63)		17 (31.48)	37 (68.52)		35 (64.81)	19 (35.19)		34 (62.96)	20 (37.04)		51 (94.44)	3 (5.56)		25 (46.30)	29 (53.70)	
**TNM stage**																			
I+II	175	99 (56.57)	76 (43.43)	0.1840	58 (33.14)	117 (66.86)	***0.0032***	122 (69.71)	53 (30.29)	0.1665	132 (75.43)	43 (24.57)	***0.0028***	160 (91.43)	15 (8.57)	0.7378	99 (56.57)	76 (43.43)	0.1840
III+IV	60	28 (46.67)	32 (53.33)		8 (13.33)	52 (86.67)		36 (60.00)	24 (40.00)		33 (55.00)	27 (45.00)		54 (90.00)	6 (10.00)		28 (46.67)	32 (53.33)	
**T stage**																			
T1+T2	107	27 (25.23)	80 (74.77)	0.5448	33 (30.84)	74 (69.16)	0.3901	76 (71.03)	31 (28.97)	0.2572	74 (69.16)	33 (30.84)	0.7467	98 (91.59)	9 (8.41)	0.7965	58 (54.21)	49 (45.79)	0.9634
T3+T4	128	28 (21.88)	100 (78.12)		33 (25.78)	95 (74.22)		82 (64.06)	46 (35.94)		91 (71.09)	37 (28.91)		116 (90.62)	12 (9.38)		69 (53.91)	59 (46.09)	
**LN invasion**																			
No	138	34 (24.64)	104 (75.36)	0.5943	42 (30.43)	96 (69.57)	0.3391	95 (68.84)	43 (31.16)	0.5314	101 (73.19)	37 (26.81)	0.2342	126 (91.30)	12 (8.70)	0.8775	76 (55.07)	62 (44.93)	0.7055
Yes	97	21 (21.65)	76 (78.35)		24 (24.74)	73 (75.26)		63 (64.95)	34 (35.05)		64 (65.98)	33 (34.02)		88 (90.72)	9 (9.28)		51 (52.58)	46 (47.42)	
**Distant metastasis**																			
M0	210	53 (25.24)	157 (74.76)	0.0543	63 (30.00)	147 (70.00)	0.0583	142 (67.62)	68 (32.38)	0.7155	151 (71.90)	59 (28.10)	0.1002	190 (90.48)	20 (9.52)	0.3601	116 (55.24)	94 (44.76)	0.2865
M1	25	2 (8.00)	23 (92.00)		3 (12.00)	22 (88.00)		16 (64.00)	9 (36.00)		14 (56.00)	11 (44.00)		24 (96.00)	1 (4.00)		11 (44.00)	14 (56.00)	

Table S3 The univariate Kaplan–Meier survival analysis for PDAC patients

Characteristics	n (125)	Discovery cohort Median Survival (months;95% CI)	*p* (KP)	n (110)	Validation cohort Median Survival (months;95% CI)	*p* (KP)	n (235)	Combined cohort Median Survival (months;95% CI)	*p* (KP)
**Age**									
<60	70	13(8.45-17.55)	0.828	43	12(8.63-19.37)	0.638	113	13(10.00-17.00)	0.6807
≥60	55	11(6.46-15.54)		67	11(7.70-15.63)		122	11(8.23-14.63)	
**Gender**									
Male	75	11(8.17-13.83)	0.208	66	11(6.92-15.22)	0.768	141	11(10.00-15.00)	0.4544
Female	50	14(2.78-25.25)		44	9(3.86-13.39)		94	12(8.63-21.03)	
**Site**									
Head	88	11(6.82-15.18)	0.557	81	11(7.20-13.81)	0.251	169	11(9.00-14.47)	0.1719
Body/tail	37	12(6.04-17.96)		29	13(1.17-24.57)		66	12(10.00-33.00)	
**Grade**									
I+II	85	18(4.10-31.90)	***<0.0001***	77	11(7.49-14.45)	0.929	181	13(10.20-18.17)	***0.0189***
III	40	12(9.12-14.88)		33	11(3.11-18.15)		54	9(7.00-14.00)	
**TNM stage**									
I+II	109	14(9.74-18.26)	***<0.0001***	66	16(9.84-21.76)	***0.001***	175	15(11.07-20.00)	***<0.0001***
III+IV	16	7(5.04-8.96)		44	6 (4.85-7.61)		60	7(5.50-9.00)	
**T stage**									
T1+T2	77	13(8.70-17.30)	0.164	30	16(11.57-20.49)	0.073	107	14(10.50-18.43)	0.5315
T3+T4	48	11(8.28-13.72)		80	8(5.14-11.52)		128	10(8.03-13.00)	
**LN invasion**									
No	76	15(3.90-26.10)	***0.013***	62	11(8.09-12.91)	0.707	138	13(10.00-23.00)	***0.0243***
Yes	49	10(7.72-12.28)		48	12(3.81-19.39)		97	11(8.00-14.00)	
**Distant metastasis**									
M0	119	13(9.19-16.82)	***0.005***	91	13(8.97-16.77)	***0.014***	210	13(10.20-15.80)	***<0.0001***
M1	6	4(2.40-5.60)		19	4(0.87-6.93)		25	4(3.47-11.60)	
**oCICs**									
No	28	27(13-NA)	***0.021***	27	30(8.37-50.69)	***0.003***	55	30 (14.63-NA)	***0.0008***
Yes	97	10(7.43-12.57)		83	9(6.80-11.86)		180	10(8.27-12.87)	
**TiT**									
No	35	21(3.84-38.16)	0.089	31	29(13.36-45.38)	***0.008***	66	21(12.27-NA)	***0.0065***
Yes	90	11(7.91-14.09)		79	9(5.83-12.83)		169	10(8.27-13.00)	
**LiT**									
No	92	14(5.54-22.46)	***0.008***	66	14(9.23-18.63)	***0.011***	158	14(10.97-21.00)	***0.0003***
Yes	33	8(6.12-9.88)		44	9(4.58-12.68)		77	9(7.00-13.00)	
**MiT**									
No	79	15(4.39-25.62)	***0.001***	86	13(8.96-16.78)	***0.003***	165	14(11.00-19.17)	***0.0004***
Yes	46	7(5.34-8.66)		24	7(1.81-12.73		70	7(6.00-10.00)	
**TiM**									
No	114	13(8.98-17.09)	0.184	100	12(7.17-16.03)	0.346	214	12(10.00-15.63)	0.1101
Yes	11	7(2.95-11.05)		10	8(2.70-12.71)		21	8(7.00-28.00)	
**L/MiT**									
No	63	31(12.27-49.73)	***<0.0001***	64	14(8.69-19.17)	***0.004***	127	15(12.27-33.00)	***<0.0001***
Yes	62	8(6.07-9.93)		46	8(5.60-10.94)		108	8(7.00-10.50)	

Table S4 Univariate analysis of prognostic parameters for survival in PDAC patients by Cox regression analysis

Characteristics	**n** (125)	Discovery cohort	***p***(COX)	**n** (110)	Validation cohort	***p***(COX)	**n**(235)	Combined cohort	***p***(COX)
		**HR** (95% CI)			**HR** (95% CI)			**HR** (95% CI)	
**Age** (≥60/<60)	55/70	0.956(0.629-1.453)	0.832	67/43	0.976(0.586-1.516)	0.807	122/113	1.020 (0.753-1.383)	0.8962
**Sex** (Female/Male)	50/75	0.764(0.497-1.174)	0.219	44/66	1.070(0.682-1.679)	0.768	94/141	0.902(0.662-1.229)	0.5135
**Site** (Body+tail/Head)	37/88	0.875(0.555-1.381)	0.567	29/81	0.726(0.419-1.258)	0.253	66/169	0.801(0.565-1.135)	0.2122
**Grade** (III/I+II)	40/85	2.215(1.403-3.311)	***<0.0001***	33/77	1.022 (0.637-1.640)	0.929	54/181	2.190(1.426-3.364)	***0.0003***
**TNM stage** (III+IV/I+II)	16/109	2.629(1.491-4.634)	***0.001***	44/66	2.029(1.302-3.160)	***0.002***	60/175	2.281(1.601-3.250)	***<0.0001***
**T stage** (T3+T4/T1+T2)	48/77	0.738(0.476-1.145)	0.169	80/30	1.572(0.954-2.589)	0.076	128/107	1.032(0.757-1.407)	0.8417
**LN invasion** (Yes/No)	49/76	1.673(1.101-2.542)	***0.016***	48/62	1.088(0.701-1.688)	0.707	97/138	1.687 (1.110-2.564)	***0.0143***
**Distant metastasis** (M1 /M0)	6/119	3.075(1.329-7.114)	***0.009***	19/91	1.975(1.133-3.441)	***0.016***	25/210	3.172 (1.372-7.336)	***0.007***
**oCICs** (Yes/No)	97/28	1.874(1.075-3.266)	***0.027***	83/27	2.431(1.313-4.499)	***0.005***	180/55	1.981(1.320-2.972)	***0.001***
**TiT** (Yes/No)	90/35	1.509(0.925-2.462)	0.099	79/31	2.063(1.190-3.573)	***0.01***	169/66	1.648(1.149-2.365)	***0.0067***
**LiT** (Yes/No)	33/92	1.798(1.149-2.812)	***0.010***	44/66	1.771(1.134-2.767)	***0.012***	77/158	1.701(1.240-2.333)	***0.001***
**MiT** (Yes/No)	46/79	1.932(1.268-2.943)	***0.002***	24/86	2.140(1.287-3.560)	***0.003***	70/165	1.971(1.294-3.002)	***0.0016***
**TiM** (Yes/No)	11/114	1.574(0.79-3.137)	0.198	10/100	1.395(0.695-2.799)	0.349	21/214	1.463(0.897-2.388)	0.1275
**L/MiT** (Yes/No)	62/63	2.249(1.470-3.441)	***<0.0001***	46/64	1.907(1.220-2.981)	***0.005***	108/127	1.984(1.464-2.688)	***<0.0001***

Table S5 AUC values calculated at different survival time points by ROC analysis

Time points	6 months		10.6 months		14 months	
L/MiT inclusion	with	w/o	with	w/o	with	w/o
**Discovery cohort**	0.737	0.657	0.760	0.676	0.767	0.71
**Validation cohort**	0.725	0.719	0.672	0.668	0.674	0.658
**Combined cohort**	0.710	0.653	0.698	0.653	0.696	0.661

w/o: without

Table S6 Multivariate analysis of prognostic parameters for PDAC stratified by TNM2

Prognostic parameters	TNM1/2 (n = 175)		TNM3/4 (n = 60)	
	HR(95%CI)	*P*	HR(95%CI)	*P*
**L/MiT** (Yes vs. No)	**2.3692**(1.642-3.418)	***<0.0001***		

Table S7 Multivariate analysis of prognostic parameters for PDAC stratified by TNM

Prognostic parameters	TNM1 (n = 65)		TNM2 (n = 110)		TNM3 (n = 35)		TNM4 (n = 25)	
	HR(95%CI)	*P*	HR(95%CI)	*P*	HR(95%CI)	*P*	HR(95%CI)	*P*
**Grade** (I+II vs. III)			**1.636** (0.965-2.776)	0.0678				
**MiT** (Yes vs. No)			**1.704** (0.906-3.205)	0.0983				
**LiT** (Yes vs. No)	**1.993** (1.622-5.521)	***0.0005***			**0.4879** (0.226-1.054)	0.0679		
**L/MiT** (Yes vs. No)			**1.904** (1.048-3.461)	***0.0346***				

Table S8 Multivariate analysis of prognostic parameters for PDAC stratified by Grade2

Prognostic parameters	Grade1/2 (n = 181)		Grade3 (n = 54)	
	HR(95%CI)	*P*	HR(95%CI)	*P*
**M stage** (Yes vs. No)	**1.9989** (1.060-3.769)	***0.0323***		
**TNM stage** (I+II vs. III+IV)	**1.7839** (1.183-2.691)	***0.0058***	**2.0366** (1.058-3.919)	***0.0332***
**L/MiT** (Yes vs. No)	**2.1933** (1.521-3.163)	***<0.0001***		

Table S9 Multivariate analysis of prognostic parameters for PDAC stratified by grade

Prognostic parameters	Grade1 (n = 36)		Grade2 (n = 145)		Grade3 (n = 54)	
	HR(95%CI)	*P*	HR(95%CI)	*P*	HR(95%CI)	*P*
**N stage** (Yes vs. No)	**0.3602** (0.145-0.894)	***0.0276***	**1.4230** (0.939-2.156)	***0.0863***		
**M stage** (Yes vs. No)	**3.7783** (1.221-11.68)	***0.0210***	**2.3037** (1.059-5.010)	***0.0353***		
**TNM stage** (I+II vs. III+IV)			**1.8931** (1.165-3.076)	***0.0100***	**2.0366** (1.058-3.919)	***0.0332***
**L/MiT** (Yes vs. No)			**2.2716** (1.517-3.402)	***0.0001***		

Table S10 Multivariate analysis of prognostic parameters for PDAC stratified by age

Prognostic parameters	<=60 (n = 113)		>60 (n = 122)	
	HR(95%CI)	*P*	HR(95%CI)	*P*
**Grade** (I+II vs. III)			**2.2319** (1.339-3.721)	***0.0021***
**N stage** (Yes vs. No)	**1.7430** (1.106-2.748)	***0.0167***		
**TNM stage** (I+II vs. III+IV)	**2.6303** (1.527-4.531)	***0.0005***	**2.3120** (1.460-3.661)	***0.0004***
**TiT** (Yes vs. No)			**2.0167** (1.101-3.694)	***0.0231***
**TiM** (Yes vs. No)			**2.0935** (1.149-3.812)	***0.0157***
**L/MiT** (Yes vs. No)	**11.6097**(3.872-34.81)	***<0.0001***		

Table S11 Multivariate analysis of prognostic parameters for PDAC stratified by sex

Prognostic parameters	Male (n = 141)		Female (n = 94)	
	HR(95%CI)	*P*	HR(95%CI)	*P*
**Grade** (I+II vs. III)	**1.7088** (1.098-2.659)	***0.0176***		
**N stage** (Yes vs. No)			**1.5112** (0.868-2.632)	0.1145
**M stage** (Yes vs. No)	**2.8502** (1.468-5.533)	***0.0020***		
**TNM stage** (I+II vs. III+IV)	**2.4337** (1.525-3.884)	***0.0002***	**2.2834** (1.227-4.249)	***0.0092***
**L/MiT** (Yes vs. No)	**1.7787** (1.177-2.688)	***0.0062***	**9.5464** (2.566-35.52)	***0.0008***

Table S12 Multivariate analysis of prognostic parameters for PDAC stratified by site

Prognostic parameters	Head (n = 169)		Body/tail (n = 66)	
	HR(95%CI)	*P*	HR(95%CI)	*P*
**Grade** (I+II vs. III)	**1.3941** (0.937-2.075)	0.1014		
**N stage** (Yes vs. No)			**2.5745** (1.334-4.968)	***0.0048***
**M stage** (Yes vs. No)			**3.4845** (1.531-7.926)	***0.0029***
**TNM stage** (I+II vs. III+IV)	**2.1329** (1.443-3.152)	***0.0001***		
**TiM** (Yes vs. No)			**2.6783** (0.988-7.263)	0.0529
**L/MiT** (Yes vs. No)	**1.8276** (1.277-2.656)	***0.0010***	**2.5390** (1.285-5.017)	***0.0073***

Table S13 Multivariate analysis of prognostic parameters for PDAC stratified by N stage

Prognostic parameters	No (n = 138)		Yes (n = 97)	
	HR(95%CI)	*P*	HR(95%CI)	*P*
**Grade** (I+II vs. III)			**1.7555** (1.055-2.921)	***0.0303***
**M stage** (Yes vs. No)	**2.3965** (1.225-4.689)	***0.0107***		
**TNM stage** (I+II vs. III+IV)			**2.4826** (1.563-3.944)	***0.0001***
**L/MiT** (Yes vs. No)	**1.9835** (1.307-3.010)	***0.0013***	**1.6502** (1.051-2.591)	***0.0295***

Table S14 Multivariate analysis of prognostic parameters for PDAC stratified by M stage

Prognostic parameters	No (n = 210)		Yes (n = 25)	
	HR(95%CI)	*P*	HR(95%CI)	*P*
**Grade** (I+II vs. III)	**1.4794** (1.007-2.173)	***0.0461***	**0.2820** (0.066-1.211)	0.0887
**N stage** (Yes vs. No)	**1.3054** (1.225-4.689)	0.1168		
**TNM stage** (I+II vs. III+IV)	**1.8578** (1.230-2.805)	***0.0032***	**inf.** (0.000-Inf)	0.9979
**L/MiT** (Yes vs. No)	**1.6514** (1.183-2.306)	***0.0032***	**inf.** (0.000-Inf)	0.9995

Table S15 Univariate analysis of tumor size as a prognostic parameter for survival in PDAC patients in the discovery cohort

Discovery cohort		Kaplan merrier univariate analysis		Cox univariate analysis		Cox univariate analysis	
Characteristics	n (%)	Median Survival (months;95% CI)	*p* values (Log rank test)	HR (95% CI)	*p* values (Cox)	HR (95% CI)	*p* values (Cox)
**Size of tumor**							
T1(≤2cm)	9 (7.2%)	20(0.000-49.218)	0.344	0.885(0.644-1.215)	0.449	0.974(0.877-1.081)	0.619
T2(≤4cm)	68 (54.4%)	11(6.223-15.777)					
T3(>4cm)	48 (38.4%)	11(8.016-13.984)		Analyzed as a categorical variable		Analyzed as a continuous variable	

Table S16 Univariate analysis of tumor size as a prognostic parameter for survival in PDAC patients in the validation cohort

Validation cohort		Kaplan merrier univariate analysis		Cox univariate analysis		Cox univariate analysis	
Characteristics	n (%)	Median Survival (months;95% CI)	*p* values (Log rank test)	HR (95% CI)	*p* values (Cox)	HR (95% CI)	*p* values (Cox)
**Size of tumor**							
T1(≤2cm)	11 (10.0%)	17.43(7.221-27.639)	0.369	1.196(0.957-1.495)	0.115	1.127(1.030-1.232)	0.009
T2(≤4cm)	19 (17.3%)	16.03(8.689-23.371)					
T3(>4cm)	41 (37.3%)	9.33(7.005-11.655)					
T4	39 (35.5%)	7.9(3.577-12.223)		Analyzed as a categorical variable		Analyzed as a continuous variable	

Table S17 Univariate analysis of tumor size as a prognostic parameter for survival in PDAC patients in the combined cohort

Combined cohort		Kaplan merrier univariate analysis		Cox univariate analysis		Cox univariate analysis	
Characteristics	n (%)	Median Survival (months;95% CI)	*p* values (Log rank test)	HR (95% CI)	*p* values (Cox)	HR (95% CI)	*p* values (Cox)
**Size of tumor**							
T1(≤2cm)	20 (8.5%)	17.430(4.365-30.495)	0.292	1.135(0.951-1.355)	0.162	1.055(0.984-1.131)	0.133
T2(≤4cm)	87 (37.0%)	14.000(10.481-17.519)					
T3(>4cm)	89 (37.9%)	10.000(8.297-11.703)					
T4	39 (16.6%)	7.900(3.577-12.223)		Analyzed as a categorical variable		Analyzed as a continuous variable	

TRIPOD checklist

Checklist of items to include when reporting a study developing or validating a multivariable prediction model for diagnosis or prognosis

Section/topic	Item	Development or validation?	Checklist item	Note
**Title and abstract**				
Title	1	D;V	Identify the study as developing and/or validating a multivariable prediction model, the target population, and the outcome to be predicted.	√
Abstract	2	D;V	Provide a summary of objectives, study design, setting, participants, sample size, predictors, outcome, statistical analysis, results, and conclusions.	√
**Introduction**				
Background and objectives	3a	D;V	Explain the medical context (including whether diagnostic or prognostic) and rationale for developing or validating the multivariable prediction model, including references to existing models.	√
	3b	D;V	Specify the objectives, including whether the study describes the development or validation of the model, or both.	√
**Methods**				
Source of data	4a	D;V	Describe the study design or source of data (e.g., randomised trial, cohort, or registry data), separately for the development and validation data sets, if applicable.	√
	4b	D;V	Specify the key study dates, including start of accrual; end of accrual; and, if applicable, end of follow-up.	√
Participants	5a	D;V	Specify key elements of the study setting (e.g., primary care, secondary care, general population) including number and location of centres.	√
	5b	D;V	Describe eligibility criteria for participants.	√
	5c	D;V	Give details of treatments received, if relevant.	√
Outcome	6a	D;V	Clearly define the outcome that is predicted by the prediction model, including how and when assessed.	√
	6b	D;V	Report any actions to blind assessment of the outcome to be predicted.	√
Predictors	7a	D;V	Clearly define all predictors used in developing the multivariable prediction model, including how and when they were measured.	√
	7b	D;V	Report any actions to blind assessment of predictors for the outcome and other predictors.	√
Sample size	8	D;V	Explain how the study size was arrived at.	√
Missing data	9	D;V	Describe how missing data were handled (e.g., complete-case analysis, single imputation, multiple imputation) with details of any imputation method.	√
Statistical analysis methods	10a	D	Describe how predictors were handled in the analyses.	√
	10b	D	Specify type of model, all model-building procedures (including any predictor selection), and method for internal validation.	√
	10c	V	For validation, describe how the predictions were calculated.	√
	10d	D;V	Specify all measures used to assess model performance and, if relevant, to compare multiple models.	√
	10e	V	Describe any model updating (e.g., recalibration) arising from the validation, if done.	√
Risk groups	11	D;V	Provide details on how risk groups were created, if done.	√
Development vs validation	12	V	For validation, identify any differences from the development data in setting, eligibility criteria, outcome, and predictors.	√
**Results**				
Participants	13a	D;V	Describe the flow of participants through the study, including the number of participants with and without the outcome and, if applicable, a summary of the follow-up time. A diagram may be helpful.	√
	13b	D;V	Describe the characteristics of the participants (basic demographics, clinical features, available predictors), including the number of participants with missing data for predictors and outcome.	√
	13c	V	For validation, show a comparison with the development data of the distribution of important variables (demographics, predictors, and outcome).	√
Model development	14a	D	Specify the number of participants and outcome events in each analysis.	√
	14b	D	If done, report the unadjusted association between each candidate predictor and outcome.	√
Model specification	15a	D	Present the full prediction model to allow predictions for individuals (i.e., all regression coefficients, and model intercept or baseline survival at a given time point).	√
	15b	D	Explain how to use the prediction model.	√
Model performance	16	D;V	Report performance measures (with CIs) for the prediction model.	√
Model updating	17	V	If done, report the results from any model updating (i.e., model specification, model performance).	√
**Discussion**				
Limitations	18	D;V	Discuss any limitations of the study (such as nonrepresentative sample, few events per predictor, missing data).	√
Interpretation	19a	V	For validation, discuss the results with reference to performance in the development data, and any other validation data.	√
	19b	D;V	Give an overall interpretation of the results, considering objectives, limitations, results from similar studies, and other relevant evidence.	√
Implications	20	D;V	Discuss the potential clinical use of the model and implications for future research	√
**Other information**				
Supplementary information	21	D;V	Provide information about the availability of supplementary resources, such as study protocol, Web calculator, and data sets.	√
Funding	22	D;V	Give the source of funding and the role of the funders for the present study.	√

[a] Items relevant only to the development of a prediction model are denoted by D, items relating solely to a validation of a prediction model are denoted by V, and items relating to both are denoted D;V. We recommend using the TRIPOD Checklist in conjunction with the TRIPOD explanation and elaboration document.

√ : followed
